# Sex-specific mediating role of serum uric acid in the association between dietary diversity and muscle strength in urban community-dwelling older adults

**DOI:** 10.3389/fpubh.2026.1877222

**Published:** 2026-08-18

**Authors:** Xixi Chu, Yuzhu Zhao, Xin Wang, Yiyao Ruan, Lulu Wang, Fang Wang, Guangyi Liu, Dayong Yu, Kaiyong Liu, Fangbiao Tao, Liang Ruan

**Affiliations:** 1 School of Public Health, Anhui Medical University/Center for Big Data and Population Health, Institute of Health and Medicine, Hefei Comprehensive National Science Center, Hefei, Anhui, China; 2 Key Laboratory of Population Health Across Life Cycle (Anhui Medical University), Ministry of Education of the People’s Republic of China, Hefei, Anhui, China; 3 NHC Key Laboratory of Study on Abnormal Gametes and Reproductive Tract, Anhui Medical University, Hefei, Anhui, China; 4 Anhui Provincial Key Laboratory of Environment and Population Health Across the Life Course/Key Laboratory of Environmental Toxicology of Anhui Higher Education Institutes, Anhui Medical University, Hefei, Anhui, China; 5 Xiyuan Street Community Health Service Center, Hefei, Anhui, China; 6 Nanqi Street Community Health Service Center, Hefei, Anhui, China

**Keywords:** dietary diversity, handgrip strength, mediation effects, muscle strength, older adults, uric acid

## Abstract

**Objectives:**

Dietary diversity is recognized as a key strategy for healthy aging, but the underlying mechanisms linking it to muscle strength, particularly those involving systemic metabolism, remain unclear. This study aimed to examine the association between dietary diversity and muscle strength and explore the potential mediating role of metabolic indicators.

**Methods:**

This cross-sectional study included 1,613 older adults (≥60 years) from the Anhui Urban Aging Cohort. Dietary diversity was assessed using a validated food frequency questionnaire to derive the dietary diversity score (DDS). Metabolic indicators—including serum uric acid (UA), the triglyceride-glucose (TyG) index, total cholesterol (TC), mean arterial pressure (MAP), and metabolic score (MS)—were measured from fasting blood samples. Muscle strength was evaluated by absolute handgrip strength (HGS), BMI-adjusted relative HGS, and low handgrip strength (LGS) defined by the Asian Working Group for Sarcopenia (AWGS) 2019 criteria. Multiple linear and logistic regression models were used to examine the associations, while restricted cubic splines characterized nonlinear relationships. Mediation analysis was performed to assess the potential mediating effect of metabolic indicators.

**Results:**

Higher DDS was significantly associated with increased absolute HGS (*β* = 0.366), increased relative HGS (*β* = 0.017), and a reduced risk of LGS (OR = 0.875). Notably, DDS was inversely associated with UA levels (*β* = −2.946). Elevated UA levels were negatively associated with relative HGS (*β* = −0.0003) and positively associated with the risk of LGS (OR = 1.002). Restricted cubic spline analysis revealed a significant adverse effect of elevated UA (>363 μmol/L) on relative HGS and LGS risk in males. Mediation analysis confirmed that UA significantly mediated the association between DDS and muscle strength, accounting for 5.64% (relative HGS) and 3.79% (LGS) of the total effect in the overall population. This mediating effect was significant only in males (proportions mediated: 9.22% and 8.67%, respectively).

**Conclusion:**

In urban community-dwelling older adults, dietary diversity benefited muscle strength and was partly mediated by serum uric acid. Notably, this mediating pathway was observed in men, whereas the null findings in women may reflect a complex association pattern. These findings underscore the necessity of considering sex-specific metabolic mechanisms in nutritional strategies against sarcopenia.

## 1 Introduction

Muscle strength serves as a critical early and modifiable marker of health status in older adults (1). For this demographic, it is not merely an expression of physical capability but also a significant predictor of preserved physiological function and reduced risk of major chronic diseases (2, 3). Handgrip strength (HGS), given its simplicity, objectivity, and high reproducibility, has become a widely used indicator (4). Lower HGS is associated with increased risks of falls, impaired cardiorespiratory function, cognitive decline, and all-cause mortality (5–8). Therefore, identifying potential determinants of muscle strength is essential for optimizing health outcomes and clinical trajectories in older adults.

Diet plays a fundamental role in muscle health (encompassing mass, strength, and function). Extensive previous research has predominantly focused on the effects of isolated foods or specific nutrients—such as protein, vitamin D, and dietary antioxidants—on muscle strength, elucidating their underlying mechanisms and intake recommendations (9, 10). However, the human diet is a complex entity, where the physiological effects are ultimately determined by synergistic and antagonistic interactions among multiple nutrients. Therefore, although a substantial amount of micronutrient-level evidence has accumulated, it remains challenging to integrate these findings into effective and feasible public health dietary guidelines. This gap highlights the necessity for holistic dietary assessment tools. The Dietary Diversity Score (DDS), a validated metric of overall diet quality and nutritional adequacy (11), provides a practical and integrative approach for epidemiological investigations into musculoskeletal outcomes (12, 13). Nevertheless, to date, the specific relationship between dietary diversity and muscle strength remains systematically underexplored.

On the other hand, impaired muscle health frequently coexists with a cluster of metabolic disorders, including dyslipidemia, insulin resistance, and hypertension (14–17). These conditions compromise muscle function through shared pathophysiological mechanisms, primarily chronic low-grade inflammation and oxidative stress (18). Chronic low-grade inflammation, driven by pro-inflammatory cytokines such as interleukin-6 (IL-6) and tumor necrosis factor-*α* (TNF-α), activates signaling pathways like nuclear factor-kappa B (NF-κB). This activation dysregulates muscle protein homeostasis by upregulating proteolysis and inhibiting insulin-like growth factor-1 (IGF-1)-mediated anabolism, thereby directly contributing to muscle atrophy and loss of strength (19, 20). Concurrently, oxidative stress, resulting from excessive reactive oxygen species (ROS) accumulation, impairs mitochondrial function, disrupts myofiber structure, and triggers cellular apoptosis and autophagy. These alterations collectively impair muscle contractility and regenerative capacity, ultimately leading to a decline in muscle strength (21). Importantly, diet is a key modulator of systemic metabolism, inflammation, and oxidative stress. Therefore, metabolic dysregulation may constitute a crucial mediating pathway linking dietary patterns to muscle strength, a hypothesis that has not been empirically quantified.

While diet and metabolic health are independently linked to muscle strength, their triadic relationship is not well characterized. Particularly, it is unclear to what extent the benefits of a diverse diet on muscle strength are explained by favorable changes in metabolic profiles. To address this, we focused on five key metabolic indicators: uric acid (UA), total cholesterol (TC), the triglyceride-glucose (TyG) index, mean arterial pressure (MAP), and metabolic score (MS). Accordingly, this study was designed (1) to assess the relationships among dietary diversity, metabolic dysregulation, and muscle strength, and (2) to further elucidate and quantify the mediating role of these metabolic risk factors in the association between dietary diversity and muscle strength.

## 2 Methods

### 2.1 Study population and data source

Data for this study were obtained from the Anhui Urban Aging Cohort (AUAC), a community-based prospective cohort established by Anhui Medical University. The AUAC focuses on community-dwelling older adults in urban areas to investigate the social, behavioral, and environmental determinants of healthy aging and chronic diseases amid rapid urbanization in China. Study sites were purposively selected from representative urban communities in Hefei City, Anhui Province, capturing variations in socioeconomic status, demographic structure, and access to social services. Participants were recruited through local community health service centers. At baseline, comprehensive data were collected, including sociodemographic characteristics, lifestyle factors, clinical measurements, biospecimens, and functional health status, with long-term follow-up ongoing. The cohort was designed to address the scarcity of high-quality, community-based evidence on the dynamics of healthy aging in rapidly urbanizing regions of China.

**Figure 1:**
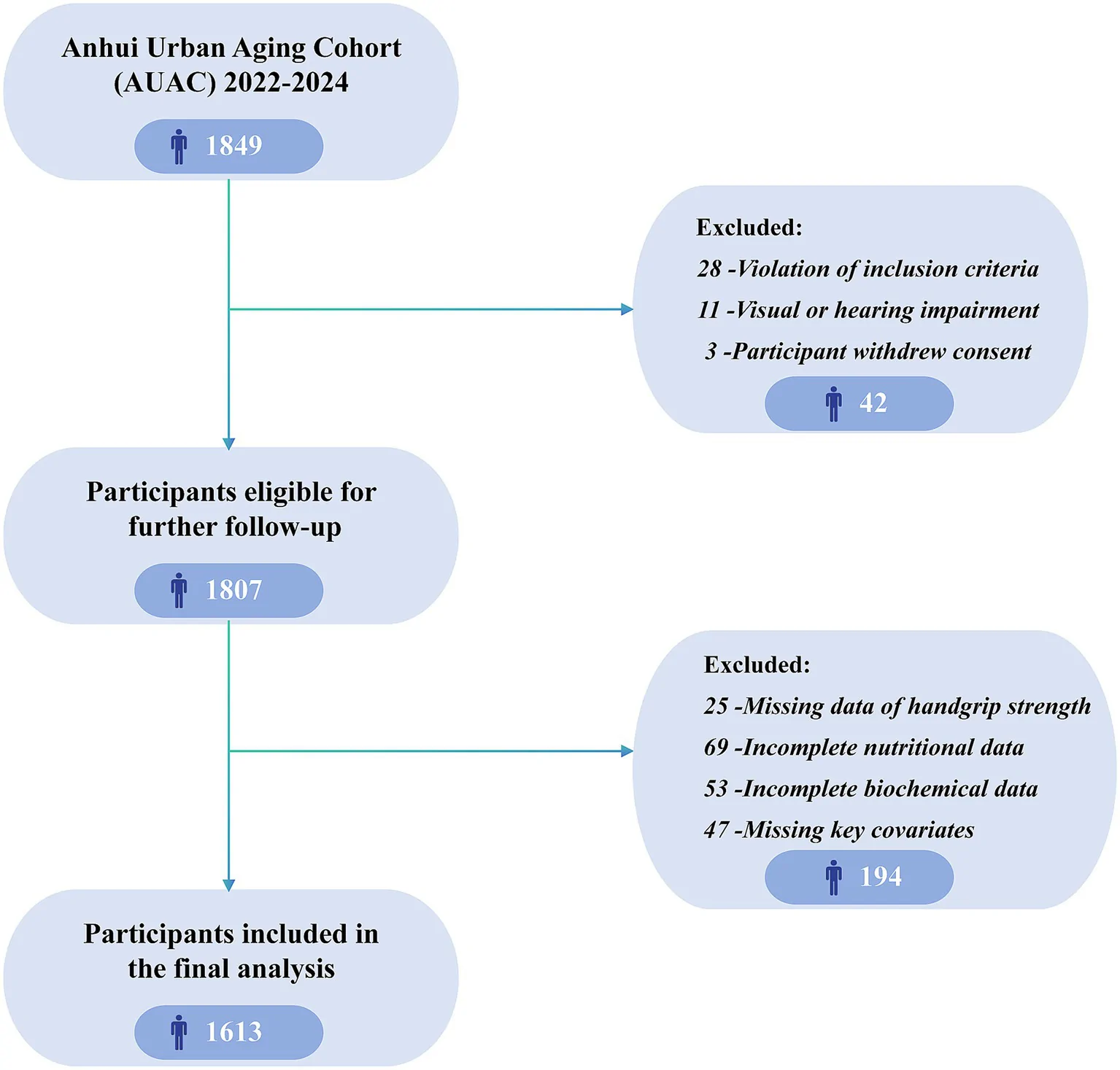
Flowchart of participant selection.

For the present study, we utilized data from a follow-up survey conducted between May 2022 and April 2024 within the AUAC cohort. The inclusion criteria were (1): age ≥ 60 years (2); continuous residence in Hefei City for at least 6 months (3); completion of a comprehensive assessment, including dietary survey, handgrip strength measurement, and blood tests for metabolic biomarkers. Exclusion criteria were: (1) history of stroke with significant limb paralysis; (2) severe arthritis or hand injuries preventing reliable handgrip measurement; (3) a diagnosis of gout or current use of urate-lowering medications; (4) severe renal insufficiency (estimated glomerular filtration rate [eGFR] < 30 mL/min/1.73 m^2^); or (5) missing data for any key variable (dietary diversity, handgrip strength, serum uric acid, or essential covariates). Furthermore, all anthropometric measurements were systematically screened for outliers and cross-checked against source documents prior to analysis. From an initial enrollment of 1,849 participants recruited from communities, 1,613 were included in the final analysis after applying the eligibility criteria (Figure 1). The study protocol was approved by the Human Research Ethics Committee of Anhui Medical University (Approval No. 20200601), and all participants provided written informed consent.

### 2.2 Assessment of dietary diversity

Dietary diversity was assessed using a 10-food-group Dietary Diversity Score (DDS) based on the Chinese Dietary Guidelines (22). At baseline, habitual consumption frequency over the past 3 months was measured using a validated food frequency questionnaire (FFQ) covering 10 major food groups: cereals, vegetables, fruits, legume products, nuts, meat, eggs, fish, dairy products, and funguses (23). One point was awarded for each food group if it was consumed at least once per week. The points were summed to yield a total DDS ranging from 0 to 10, with higher scores indicating greater dietary variety. This frequency-based scoring algorithm is consistent with established dietary diversity assessment protocols, which prioritize the breadth of food group consumption over portion size quantification (23, 24).

### 2.3 Measurement of muscle strength

Muscle strength was assessed using three metrics: handgrip strength (HGS), relative HGS (HGS/BMI), and low handgrip strength (LGS). HGS (in kg) was measured by a digital hand dynamometer (CAMRY, EH101) with the participant in a seated position, the elbow flexed at 90°, and the forearm in a neutral position. Each participant performed maximal voluntary contractions alternately with both hands, with at least two trials per hand and a 60-s rest interval between trials. The highest value obtained from the dominant hand was used as the representative HGS for analysis. Relative HGS was calculated as HGS divided by body mass index (BMI, kg/m^2^) (25, 26). LGS was defined according to the 2019 criteria of the Asian Working Group for Sarcopenia (AWGS), with cut-off values of <28 kg for men and <18 kg for women (27).

### 2.4 Assessment of metabolic indicators

This study assessed five core metabolic risk factors: mean arterial pressure (MAP), the triglyceride-glucose (TyG) index, total cholesterol (TC), uric acid (UA) and metabolic score (MS score). These indicators have been widely used to evaluate cardiometabolic risk and are commonly employed as key markers of metabolic health in large-scale epidemiological studies. All blood samples were collected after an overnight fast of at least 8 h. Fasting plasma glucose (FPG), serum creatinine, triglycerides (TG), TC, and UA were analyzed using standard enzymatic methods on an automated biochemistry analyzer (BS-830S, Mindray Medical International Limited, Shenzhen, China). Specifically, FPG was measured with the glucose oxidase method; serum creatinine was measured using an enzymatic assay based on the sarcosine oxidase method; TG, TC, and UA were determined by enzymatic colorimetric assays (GPO-PAP, CHOD-PAP, and uricase–peroxidase methods, respectively). Seated systolic and diastolic blood pressure (SBP, DBP) were measured three times using a validated automatic oscillometric monitor (Omron HEM-1020, Dalian, China) after a 5-min rest; the average of the second and third readings was used for analysis.

MAP and the TyG index (28–30) were calculated as follows:


MAP=DBP+1/3(SBP−DBP)

TyGindex=Ln[TG(mg/dL)×FPG(mg/dL)/2]

The composite MS score was derived by summing the z-scores of its four components (29): MAP, the TyG index, TC, and UA. All laboratory procedures adhered to the standardized protocols of the clinical laboratory.

### 2.5 Covariates

Covariates were selected based on previous literature and included demographic, socioeconomic, and health-related factors. Demographic and socioeconomic factors comprised: age (years, continuous), sex (male/female), educational attainment (primary school or below, junior high school, senior high school, and college or above), per capita monthly household income (low [<2,000 CNY], middle [2,000–4,999 CNY], or high [≥5,000 CNY]), marital status (married vs. others [never unmarried, divorced, separated, widowed]), and living arrangement (living alone vs. not living alone). Health-related factors included: body mass index (BMI, kg/m^2^; continuous), waist circumference (cm; continuous), smoking status (never, former, current), and alcohol consumption (never, former, current). Physical activity level was assessed based on self-reported engagement in vigorous, moderate, and light activities and categorized as: High (vigorous activities on at least 3 days/week), Middle (no vigorous but moderate activities on at least 3 days/week), or Low (all others). Estimated glomerular filtration rate (eGFR, mL/min/1.73 m^2^; continuous), calculated using the 2021 Chronic Kidney Disease Epidemiology Collaboration (CKD-EPI) creatinine equation (31), was included as a covariate to account for renal function. Multimorbidity was defined as the coexistence of two or more chronic conditions. The conditions considered were: hypertension, type 2 diabetes, dyslipidemia, coronary heart disease, stroke, chronic obstructive pulmonary disease, non-alcoholic fatty liver disease, and chronic kidney disease. The presence of these conditions was ascertained through a combination of self-report and verification against medical records. Participants were stratified into three groups based on the number of conditions: 0, 1–2, and ≥3.

### 2.6 Statistical analysis

Statistical analyses were performed using SPSS version 27.0 and R version 4.4.3. Descriptive statistics are presented as mean ± standard deviation for continuous variables and as number (percentage) for categorical variables. Differences in baseline characteristics between participants with and without low grip strength (LGS) were compared using Student’s t-test or the Chi-square test, as appropriate.

Spearman correlation analyses were first used to assess the associations between dietary diversity score (DDS), metabolic indicators, and muscle strength measures. Subsequently, multiple linear regression models were used to examine the adjusted associations of DDS with continuous outcomes, including absolute HGS, relative HGS, and the five metabolic indicators (UA, TC, TyG index, MAP, and MS). For the binary outcome (LGS), binary logistic regression was employed. Three sequential regression models were constructed. Model 1 was unadjusted; Model 2 was adjusted for age and sex; Model 3 was further adjusted for educational attainment, marital status, living alone, income, smoking status, alcohol consumption, physical activity, and eGFR. For metabolic indicators and LGS, Model 3 additionally included multimorbidity and BMI. For HGS and relative HGS, Model 3 did not include multimorbidity or BMI to avoid over-adjustment. Results are reported as *β* coefficients with 95% confidence intervals (CIs) for linear models and as odds ratios (ORs) with 95% CIs for logistic models.

To characterize the dose–response relationships between serum uric acid (UA) and the three muscle strength measures (HGS, relative HGS, and LGS), we fitted restricted cubic spline (RCS) regression models. Analyses were performed in the overall study population and were also stratified by sex. The optimal number and positions of knots for each model were selected based on the lowest Akaike Information Criterion (AIC). The overall and non-linear associations of UA with the outcomes were assessed using likelihood ratio tests. The results are presented graphically. Subsequently, mediation analyses were conducted to estimate the indirect effect of UA on the associations between DDS and the muscle strength outcomes. The magnitude of the indirect (mediating) effect was quantified using 5,000 bootstrap resamples, and its statistical significance was determined by examining whether the bias-corrected 95% bootstrap CI excluded zero. The proportion mediated was calculated as the ratio of the indirect effect to the total effect. All mediation analyses were also stratified by sex. To test the robustness of the main estimates, we performed sensitivity analyses by re-defining low handgrip strength (LGS) using the alternative cut-offs recommended by the European Working Group on Sarcopenia in Older People (EWGSOP2; <27 kg for men and <16 kg for women) (32). These sensitivity models employed the same multivariable adjustment strategy as the primary analyses. A two-tailed *p*-value < 0.05 was considered statistically significant for all analyses.

## 3 Results

### 3.1 Baseline characteristics of the study population

A total of 1,613 participants (mean age 70.89 ± 6.42 years; 41.1% male) were included. Based on sex-specific cutoffs, 441 participants (27.3%) were classified as having low grip strength (LGS), with comparable prevalence between men (27.8%) and women (27.1%). Baseline characteristics stratified by LGS status are presented in Table 1. Demographically, participants with LGS were significantly older, had lower educational attainment, lower income, a lower proportion of married individuals, and a higher rate of living alone compared to their counterparts with normal grip strength (all *p* < 0.05). Regarding health behaviors, the LGS group reported lower levels of physical activity (*p* < 0.001) and had a significantly lower dietary diversity score (DDS: 6.62 ± 1.64 vs. 6.97 ± 1.49, *p* < 0.001). No significant between-group differences were observed in smoking or drinking status. In terms of health and metabolic profiles, individuals with LGS had a greater burden of multimorbidity (*p* < 0.001), lower BMI (24.13 ± 3.55 vs. 24.53 ± 3.30 kg/m^2^, *p* = 0.041), lower TC levels (4.81 ± 1.06 vs. 5.01 ± 1.07 mmol/L, *p* < 0.001), and lower estimated glomerular filtration rate (eGFR: 86.06 ± 14.81 vs. 87.80 ± 13.57 mL/min/1.73 m^2^, *p* = 0.025), but higher serum UA levels (344.61 ± 97.91 vs. 333.92 ± 89.65 μmol/L, *p* = 0.046). Waist circumference (WC), the triglyceride-glucose (TyG) index, mean arterial pressure (MAP), and metabolic score (MS) did not differ significantly between groups.

**Table 1:** Baseline characteristics of participants by LGS status.

Variables	Total (*n* = 1,613)	LGS status		*p-*value
		No (*n* = 1,172)	Yes (*n* = 441)	
Age	70.89 ± 6.42	69.84 ± 5.94	73.67 ± 6.79	**<0.001**
Sex, *n* (%)				0.756
Male	663 (41.10)	479 (40.87)	184 (41.72)	
Female	950 (58.90)	693 (59.13)	257 (58.28)	
Educational attainment, *n* (%)				**<0.001**
Primary school and below	512 (31.74)	334 (28.50)	178 (40.36)	
Junior high school	467 (28.95)	351 (29.95)	116 (26.30)	
Senior high school	470 (29.14)	363 (30.97)	107 (24.26)	
Junior college and above	164 (10.17)	124 (10.58)	40 (9.07)	
Marital status, *n* (%)				**<0.001**
Married	1,371 (85.00)	1,018 (86.86)	353 (80.05)	
Others	242 (15.00)	154 (13.14)	88 (19.95)	
Living alone, *n* (%)				**0.040**
Yes	151 (9.36)	99 (8.45)	52 (11.79)	
No	1,462 (90.64)	1,073 (91.55)	389 (88.21)	
Per capita income, *n* (%)				**<0.001**
Low	340 (21.08)	222 (18.94)	118 (26.76)	
Middle	1,109 (68.75)	840 (71.67)	269 (60.99)	
High	164 (10.17)	110 (9.39)	54 (12.24)	
Multimorbidity, *n* (%)				**<0.001**
0	495 (30.69)	387 (33.02)	108 (24.49)	
1–2	923 (57.22)	658 (56.14)	265 (60.09)	
≥3	195 (12.09)	127 (10.84)	68 (15.42)	
Smoking status, *n* (%)				0.438
Current	192 (11.90)	146 (12.46)	46 (10.43)	
Former	204 (12.65)	151 (12.88)	53 (12.02)	
Never	1,217 (75.45)	875 (74.66)	342 (77.55)	
Alcohol consumption, *n* (%)				0.566
Current	223 (13.83)	168 (14.33)	55 (12.47)	
Former	210 (13.02)	149 (12.71)	61 (13.83)	
Never	1,180 (73.16)	855 (72.95)	325 (73.70)	
Physical activity, *n* (%)				**<0.001**
Low	242 (15.00)	153 (13.05)	89 (20.18)	
Middle	1,268 (78.61)	929 (79.27)	339 (76.87)	
High	103 (6.39)	90 (7.68)	13 (2.95)	
WC (cm)	86.59 ± 10.00	86.66 ± 9.94	86.40 ± 10.17	0.647
BMI (kg/m^2^)	24.42 ± 3.37	24.53 ± 3.30	24.13 ± 3.55	**0.041**
UA (μmol/L)	336.85 ± 92.07	333.92 ± 89.65	344.61 ± 97.91	**0.046**
TC (mmol/L)	4.95 ± 1.07	5.01 ± 1.07	4.81 ± 1.06	**<0.001**
TyG index	8.75 ± 0.57	8.76 ± 0.57	8.74 ± 0.57	0.554
MAP (mmHg)	96.59 ± 11.27	96.55 ± 11.20	96.70 ± 11.46	0.810
MS	−0.00 ± 2.22	0.02 ± 2.18	−0.07 ± 2.32	0.468
eGFR (ml/min/1.73m^2^)	87.32 ± 13.94	87.80 ± 13.57	86.06 ± 14.81	**0.025**
DDS	6.88 ± 1.54	6.97 ± 1.49	6.62 ± 1.64	**<0.001**
Relative HGS(kg/BMI)	1.05 ± 0.36	1.16 ± 0.34	0.76 ± 0.26	**<0.001**

Data are presented as mean ± standard deviation for continuous variables and number (percentage) for categorical variables. Continuous and categorical variables were compared using the independent samples t-test and the Chi-squared test, respectively. All numerical values are rounded to two decimal places; negative values retain their sign even if rounded to zero. Bold values indicate statistical significance (P < 0.05). All other variable abbreviations are as defined in the main text.

### 3.2 Correlations between DDS, metabolic indicators, and measures of muscle strength

**Figure 2:**
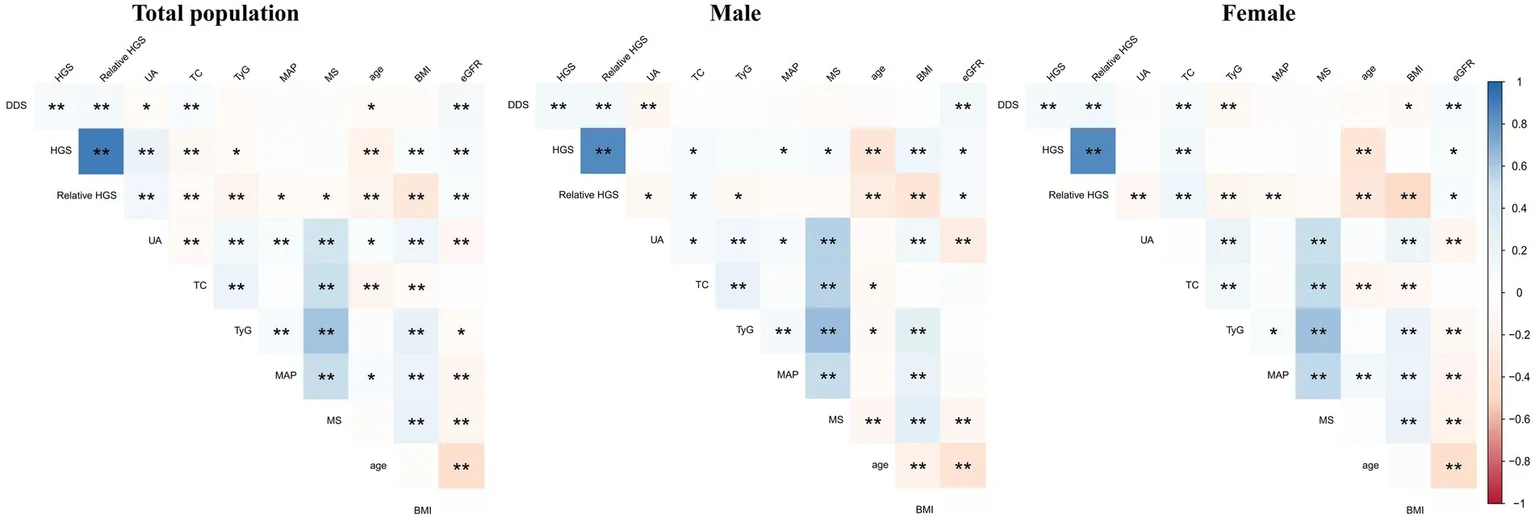
Correlation coefficient matrices for health-related indicators, stratified by sex. Panels display lower-triangular correlation heatmaps for all participants, males, and females from left to right. Only statistically significant correlations are displayed by asterisks (^*^p < 0.05, ^**^p < 0.01). Non-significant associations are left blank. Correlation coefficients (*r*) are represented by a color gradient (blue: positive, red: negative) as indicated by the scale bar. Variable abbreviations are defined in the main text.

Correlation analyses among DDS, metabolic indicators, and measures of muscle strength are presented in Figure 2. In the overall study population, DDS was positively associated with both HGS (*r* = 0.08) and relative HGS (*r* = 0.10). Regarding metabolic indicators, DDS was positively correlated with total cholesterol (TC: *r* = 0.07) and inversely correlated with serum uric acid (UA: *r* = −0.05). Notably, while relative HGS was inversely correlated with most metabolic indicators, it exhibited a positive association with UA in the overall study population (*r* = 0.15). However, sex-stratified analyses revealed a reversal of this association: relative HGS was negatively correlated with UA in both men (*r* = −0.08) and women (*r* = −0.10). For completeness, exact correlation coefficients and statistical significance levels for all bivariate associations are reported in Supplementary Tables S1–S3.

### 3.3 Associations among DDS, metabolic indicators, and measures of muscle strength

**Figure 3:**
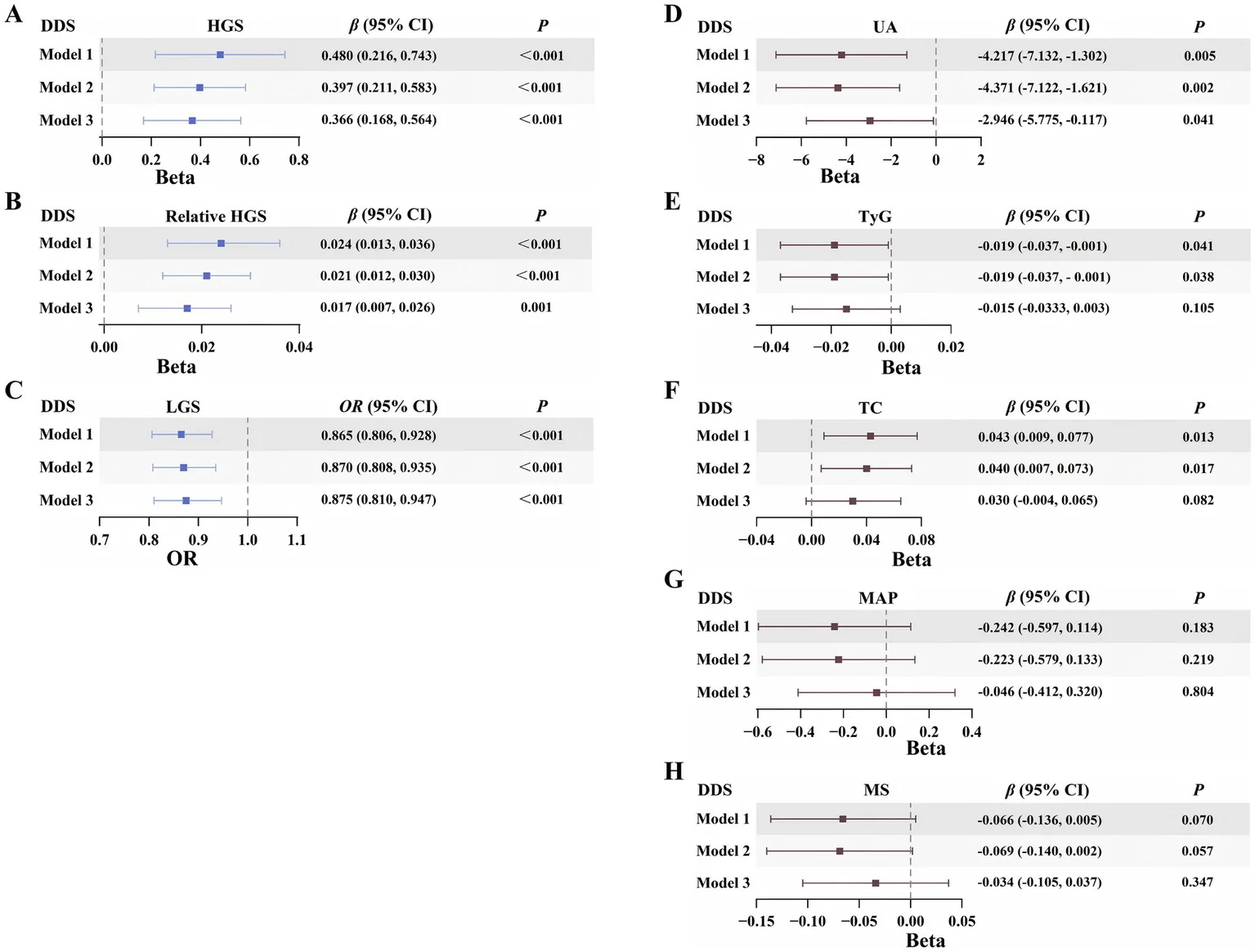
Associations of dietary diversity score (DDS) with muscle strength and metabolic indicators. **(A–C)** Associations with measures of muscle strength: HGS, relative HGS, and LGS. **(D–H)** Associations with metabolic indicators: UA, TyG index, TC, MAP, and MS. Forest plots display the *β* coefficients (for continuous outcomes: HGS, relative HGS, and metabolic indicators) or odds ratios (*OR*, for binary outcomes: LGS) with 95% confidence intervals (*CI*) across three sequential adjustment models: Model 1: Unadjusted; Model 2: Adjusted for age, sex; Model 3: **(A,B)** Were adjusted for age, sex, educational attainment, marital status, living alone, income, smoking status, alcohol consumption, physical activity, and *eGFR*. **(C–H)** Were adjusted for all covariates in Model 2 plus multimorbidity and BMI. Square markers and horizontal lines represent point estimates and 95% CIs, respectively. The vertical dashed line indicates the null effect (*β* = 0 for continuous outcomes; OR = 1 for LGS). All other variable abbreviations are as defined in the main text.

Across all models (Models 1–3), DDS showed significant positive associations with HGS and relative HGS, and a significant negative association with the risk of LGS (all *p* < 0.05; Figures 3A–C). In the fully adjusted model (Model 3), higher DDS was significantly associated with increased HGS (*β* = 0.366, 95% CI: 0.168, 0.564; *p* < 0.001) and relative HGS (*β* = 0.017, 95% CI: 0.007, 0.026; *p* = 0.001), and with a lower risk of LGS (OR = 0.875, 95% CI: 0.810, 0.947; *P* = < 0.001).

In Model 3, DDS was inversely associated with UA (*β* = −2.946, 95% CI: −5.775, −0.117; *p* = 0.041; Figure 3D) but showed no significant associations with the other four metabolic indicators (Figures 3E–H).

Among all metabolic indicators, UA was the only one significantly associated with both relative HGS and LGS in Model 3. Higher UA levels were inversely associated with relative HGS (*β* = −0.0003, 95% CI: −0.0005, −0.0002; *p* < 0.001) and positively associated with the risk of LGS (OR = 1.002, 95% CI: 1.001, 1.003; *p* = 0.004). Other metabolic indicators also showed significant associations with muscle strength in Model 3: TC, MAP, and MS were associated with HGS, while TC, TyG, MAP, and MS were associated with relative HGS (all *p* < 0.05). Detailed coefficients for all associations are presented in Table 2.

**Table 2:** Associations of metabolic indicators with muscle strength measures.

Metabolic indicators	HGS		Relative HGS		LGS	
	*β* (95% CI)	*p*-value	*β* (95% CI)	*p*-value	OR (95% CI)	*p*-value
Model 1						
UA	0.019 (0.015, 0.023)	**<0.001**	0.001 (0.0003, 0.001)	**<0.001**	1.001 (1.001, 1.002)	**0.038**
TC	−0.704 (−1.081, −0.328)	**<0.001**	−0.020 (−0.037, −0.003)	**0.018**	0.838 (0.755, 0.930)	**<0.001**
TyG	−0.660 (−1.370, 0.050)	0.069	−0.095 (−0.126, −0.064)	**<0.001**	0.944 (0.779, 1.144)	0.554
MAP	0.022 (−0.014, 0.058)	0.237	−0.002 (−0.003, −0.0002)	**0.028**	1.001 (0.992, 1.011)	0.810
MS	0.179 (−0.004, 0.362)	0.056	−0.010 (−0.018, −0.002)	**0.020**	0.982 (0.934, 1.032)	0.468
Model 2						
UA	−0.001 (−0.004, 0.003)	0.653	−0.0003 (−0.0004, −0.0001)	**<0.001**	1.001 (1.001, 1.003)	**0.035**
TC	0.379 (0.101, 0.657)	**0.008**	0.026 (0.013, 0.039)	**<0.001**	0.881 (0.788, 0.985)	**0.026**
TyG	0.296 (−0.209, 0.802)	0.250	−0.057 (−0.081, −0.033)	**<0.001**	0.965 (0.790, 1.178)	0.725
MAP	0.033 (0.007, 0.059)	**0.012**	−0.001 (−0.003, −0.0001)	**0.030**	0.998 (0.988, 1.008)	0.730
MS	0.178 (0.048, 0.307)	**0.007**	−0.009 (−0.015, −0.003)	**0.003**	0.990 (0.941, 1.042)	0.697
Model 3						
UA	−0.002 (−0.005, 0.002)	0.309	−0.0003 (−0.0005, −0.0002)	**<0.001**	1.002 (1.001, 1.003)	**0.004**
TC	0.369 (0.088, 0.649)	**0.010**	0.024 (0.011, 0.037)	**<0.001**	0.893 (0.796, 1.000)	0.051
TyG	0.258 (−0.251, 0.767)	0.320	−0.059 (−0.082, −0.036)	**<0.001**	0.994 (0.804, 1.227)	0.957
MAP	0.029 (0.003, 0.055)	**0.029**	−0.002 (−0.003, −0.0003)	**0.014**	1.000 (0.989, 1.010)	0.980
MS	0.145 (0.013, 0.277)	**0.032**	−0.011 (−0.017, −0.005)	**<0.001**	1.009 (0.956, 1.064)	0.754

Data are β (95% CI) for continuous outcomes (HGS, relative HGS) and OR (95% CI) for LGS. Model 1: Unadjusted. Model 2: Adjusted for age and sex. Model 3: Adjusted for age, sex, educational attainment, marital status, living alone, income, smoking status, alcohol consumption, physical activity, and eGFR. Additionally, models for LGS were further adjusted for multimorbidity and BMI. Bold values indicate statistical significance (*p* < 0.05). Variable abbreviations are defined in the main text.

### 3.4 Associations of UA with multiple muscle strength measures

**Figure 4:**
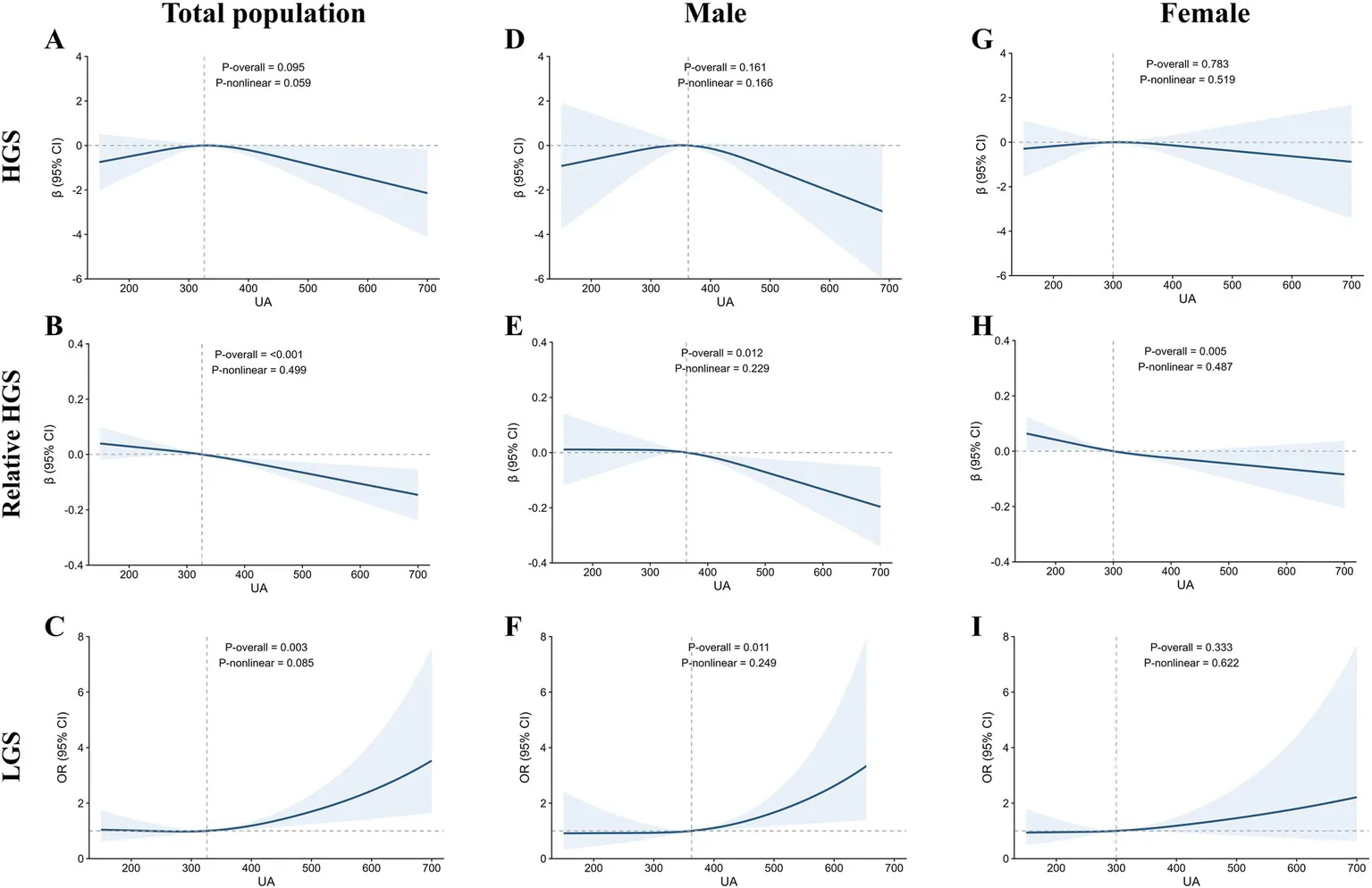
Dose–response associations of UA with muscle strength measures, stratified by sex. Restricted cubic spline plots depict the associations of serum uric acid with absolute HGS, relative HGS, and LGS in the total population, males, and females. Rows represent the outcomes of **(A,D,G)** absolute HGS, **(B,E,H)** relative HGS, and **(C,F,I)** low handgrip strength (LGS). Columns represent the total population **(A–C)**, males **(D–F)**, and females **(G–I)**. Models for the HGS and relative HGS groups were adjusted for age, sex, educational attainment, marital status, living alone, income, smoking status, alcohol consumption, physical activity, and eGFR. Models for the LGS group were further adjusted for multimorbidity and BMI. In the sex-stratified analyses, sex was used as a stratification variable, not a covariate. The solid lines represent the adjusted *β* coefficients (for continuous HGS and relative HGS) or odds ratios (for binary LGS). The dashed horizontal line indicates the null effect (*β* = 0 or *OR* = 1). The shaded areas denote 95% confidence intervals. *p* values for the overall association and for nonlinearity are shown within each panel.

UA levels showed distinct associations with different measures of muscle strength (Figure 4). No significant association was observed between UA and absolute handgrip strength (HGS) in any group (all *P* for overall > 0.05) (Figures 4A,D,G). In contrast, a significant linear association was found between UA and relative HGS after adjustment for confounders. UA was associated with relative HGS in the total population (*P* for overall < 0.001, *P* for nonlinearity = 0.499), in males (*P* for overall = 0.012, *P* for nonlinearity = 0.229), and in females (*P* for overall = 0.005, *P* for nonlinearity = 0.487) (Figures 4B,E,H).

Sex-stratified RCS curves revealed that in males, a significant inverse association between UA and relative HGS emerged at higher UA levels (approximately > 363 μmol/L) (Figure 4E), whereas in females, the curve suggested a positive association at lower UA levels (approximately < 300 μmol/L), with no significant association above this threshold (Figure 4H). Similarly, a significant linear association was observed between UA and the risk of low handgrip strength (LGS) in the total population (*P* for linearity = 0.003, *P* for nonlinearity = 0.085) and in males (*P* for linearity = 0.011, *P* for nonlinearity = 0.249), but not in females (Figures 4C,F,I). In males, the curve indicated that the risk of LGS increased significantly at UA levels exceeding 363 μmol/L (Figure 4F).

### 3.5 Mediating role of UA in the association between DDS and muscle strength

**Figure 5:**
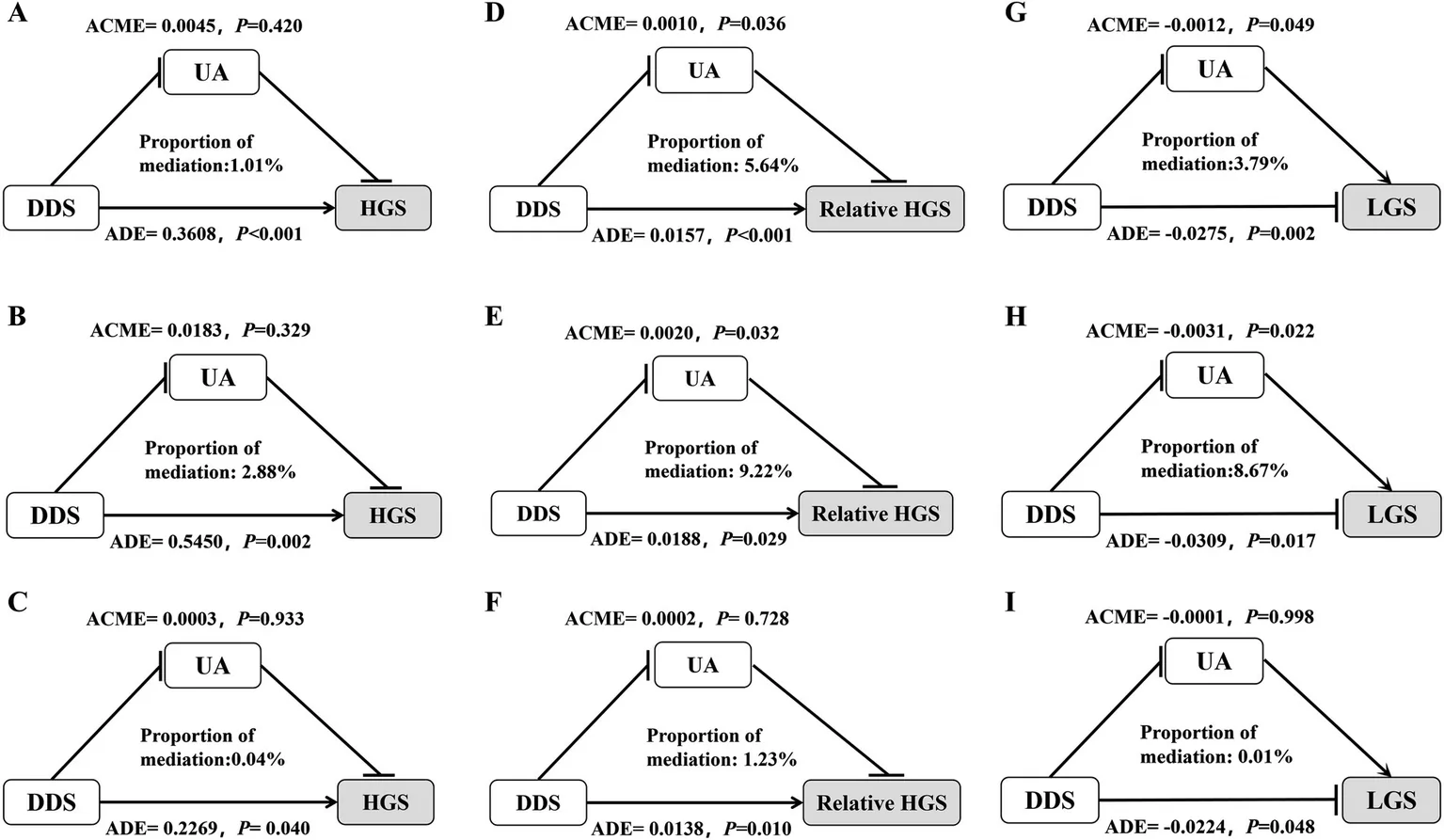
Mediation effects of UA on the associations between DDS and muscle strength measures, stratified by sex. Rows represent: **(A,D,G)** All participants; **(B,E,H)** Male participants; **(C,F,I)** Female participants. Columns represent: **(A–C)** Absolute HGS; **(D–F)** Relative HGS; **(G–I)** Low handgrip strength (LGS). ACME (average causal mediation effect) indicates the indirect effect via UA; ADE (average direct effect) indicates the direct effect of DDS on muscle strength. Proportions represent the percentage of the total effect mediated by UA. *p* values indicate the statistical significance of ACME and ADE. Solid arrows denote promotion, and rounded heads denote inhibition. Models for the HGS and relative HGS groups were adjusted for age, sex, educational attainment, marital status, living alone, income, smoking status, alcohol consumption, physical activity, and eGFR. Models for the LGS group were further adjusted for multimorbidity and BMI. In the sex-stratified analyses, sex was used as a stratification variable, not a covariate.

Mediation analyses were performed to examine the potential mediating role of serum uric acid (UA) in the associations between dietary diversity score (DDS) and muscle strength indicators (Figure 5). In the total population, UA exhibited a significant mediating effect in the associations of DDS with both relative HGS and LGS, accounting for 5.64% and 3.79% of the total effects, respectively (Figures 5D,G). In contrast, UA did not serve as a significant mediator in the association between DDS and absolute HGS (Figure 5A).

Sex-specific analyses revealed distinct patterns. Among males, UA significantly mediated the associations of DDS with relative HGS and LGS, with higher proportions of mediation (9.22% and 8.67%, respectively; Figures 5E,H). No significant mediation was observed for the DDS–HGS association in males (Figure 5B), which was consistent with the overall findings. Conversely, among females, UA did not demonstrate a significant mediating effect in any of the associations between DDS and the muscle strength indicators (HGS, relative HGS, or LGS; all *p* > 0.05). Detailed statistical estimates are provided in Supplementary Table S4.

### 3.6 Sensitivity analyses

In a sensitivity analysis applying the EWGSOP2 criteria to define low handgrip strength, the observed associations remained largely consistent with the main findings. Specifically, the sex-specific pattern of UA mediation persisted, with a significant indirect effect in men but no significant linear mediation detected in women, corroborating the robustness of our results across commonly used sarcopenia grip-strength thresholds (Supplementary Tables S5, S6; Supplementary Figure S1).

## 4 Discussion

Based on a representative community-based cohort of older adults, this study systematically investigated the relationship between dietary diversity and muscle strength, and comprehensively evaluated the potential role of metabolic risk factors. Our findings confirm that a higher dietary diversity score (DDS) is associated with better muscle strength, and serum uric acid (UA) acts as a significant sex-specific mediator in this association. Specifically, in the overall population, UA mediated 5.64% and 3.79% of the associations between DDS and relative handgrip strength (HGS) and between DDS and low muscle strength (LGS), respectively. However, this mediating effect was statistically significant only in men, with mediation proportions of 9.22% and 8.67%, respectively. Furthermore, the association between UA and muscle strength also exhibited a sex-specific pattern. In males, UA levels exceeding 363 μmol/L were significantly associated with decreased relative HGS and an increased risk of LGS. In contrast, in females, a positive association with relative HGS was observed only at lower uric acid levels (approximately < 300 μmol/L), with no significant correlation detected above this threshold. These findings reveal a novel pathway through which dietary diversity influences muscle strength by modulating UA metabolism, and provide a basis for targeted nutritional interventions.

This study employed a multidimensional set of muscle strength indicators, confirming that a higher dietary diversity score is associated with greater absolute HGS and relative HGS, as well as a lower risk of LGS. This finding is consistent with existing literature and further underscores the importance of dietary diversity as a core dietary principle for healthy aging (33). The observed associations can be explained through several interrelated mechanisms. On the one hand, a diverse diet facilitates comprehensive access to proteins and essential amino acids necessary for maintaining muscle anabolism. Recent evidence demonstrates that animal- and plant-derived proteins exhibit comparable efficacy in promoting muscle synthesis (34, 35). On the other hand, a diverse dietary pattern rich in fruits, vegetables, and whole grains provides abundant antioxidant nutrients and bioactive compounds, which collectively enhance endogenous antioxidant defenses (36–38). This, in turn, helps mitigate age-related oxidative stress—a key driver of the decline in muscle strength and function. Collectively, these insights provide a plausible explanation for the association between DDS and muscle strength.

Our study further reveals that serum uric acid is a significant mediator in the association between dietary diversity and muscle strength. Our results suggest that optimizing dietary structure (e.g., by increasing dietary diversity) may preserve muscle health not only through direct nutritional pathways but also indirectly by improving systemic metabolic homeostasis, specifically by modulating serum uric acid levels. While prior research has predominantly focused on direct factors such as protein and vitamin D (39), our findings provide a novel metabolic perspective on nutrition and muscle health. We propose that the underlying mechanism may involve an integrated metabolic pathway. Given that accumulating evidence positions UA not merely as a biomarker but as an active participant in metabolic syndrome (40, 41), a diversified diet could promote UA excretion and lower its serum levels through multiple means, including improved insulin sensitivity, reduced chronic low-grade inflammation, and modulation of the gut microbiota and its metabolites (e.g., short-chain fatty acids) (42–45). The subsequent reduction in circulating UA may, in turn, help mitigate oxidative damage to muscle mitochondria, thereby supporting more efficient energy metabolism for muscle contraction (44, 46). Thus, uric acid can be regarded as a critical metabolic node linking dietary patterns, systemic metabolism, and target-tissue (i.e., muscle) strength.

A notable finding of this study is the sex-specific nature of the mediating effect of UA, which was significant only in men for the association between dietary diversity and muscle strength. This disparity can be attributed to a convergence of physiological, hormonal, and metabolic factors. First, at the physiological level, men typically possess greater muscle mass accompanied by more active purine metabolism, which may result in a closer association between circulating UA levels and skeletal muscle status (47). Second, and perhaps most critically, divergent hormonal environments lead to sex-specific biological responses to UA involving oxidative stress, chronic low-grade systemic inflammation, and skeletal muscle mitochondrial dysfunction (48, 49). In older men, elevated serum UA triggers a continuous linear pathological cascade of oxidative injury, persistent inflammatory signaling, and impaired mitochondrial energy metabolism, ultimately accelerating muscle strength loss. In contrast, postmenopausal older women benefit from the lasting protective imprint of long-term premenopausal estrogen exposure, alongside residual endogenous estrogen. Together, these hormonal factors deliver inherent antioxidant and anti-inflammatory properties to counterbalance UA-mediated metabolic damage. This dual hormonal protective buffer interrupts the linear mediating pathway of UA, leading to statistically non-significant indirect mediation effects in females, which may reflect a more complex association pattern. Third, differences in disease comorbidity profiles further amplify this sex disparity. Among older men, hyperuricemia often coexists with muscle-harming metabolic disorders including visceral adiposity and insulin resistance (50–54). Accordingly, serum UA in men acts not only as a routine biochemical marker but also a robust indicator of sustained systemic metabolic dysregulation. Collectively, these factors position UA as a prominent metabolic intermediary linking diet to muscle strength, particularly in men.

Furthermore, dose–response analyses elucidated the nuanced association patterns between UA and muscle strength, providing a mechanistic basis for the observed sex disparity. The identified threshold in men (~363 μmol/L) lies substantially below the conventional diagnostic cutoff for hyperuricemia (420 μmol/L). This observation aligns with the established dual biological role of UA, which exerts antioxidant properties at low physiological concentrations but shifts to pro-oxidant and pro-inflammatory effects when elevated (55, 56). Consequently, the mediating effect of UA reflects the avoidance of the transition into the high-risk, pro-oxidant range, rather than a universal benefit of indiscriminate UA reduction. Indeed, the differential associations across sexes underscore this complexity. In men, the harm observed at levels >363 μmol/L suggests a predominance of pro-inflammatory injury effects. Conversely, the potential benefit observed at lower UA levels (<300 μmol/L) in women likely reflects the antioxidant capacity. Given this potential U-shaped association—wherein UA exhibited a moderate positive association with relative HGS at lower concentrations but a null association at higher levels—the linear mediation model employed in this study may have lacked statistical power to discern subtle effects, particularly given the relatively low proportion of men with low UA levels (~20%). This necessitates cautious interpretation of these null findings pending validation in larger, sex-stratified prospective studies.

While this finding suggests that the metabolic burden of UA may commence within the so-called “normal-high” range (e.g., 363–419 μmol/L), it should be interpreted strictly as an empirically observed, sex-specific signal rather than an immediate basis for clinical intervention guidelines. Nevertheless, this lower-than-conventional threshold (~363 μmol/L) highlights the feasibility of adopting differentiated UA cut-offs tailored for older adults. Such an approach could serve as a cost-effective tool for the early risk stratification of sarcopenia within the framework of routine geriatric health examinations. By identifying older men whose UA levels enter the high-risk range before the manifestation of overt muscle impairment, clinicians may initiate timely nutritional or lifestyle counseling. Definitive translation into clinical practice, however, must be preceded by validation in large-scale, prospective cohorts.

Our study established a multi-dimensional muscle strength assessment system that encompassed absolute HGS, relative HGS (HGS/BMI), and LGS. A key observation was that the mediating effect of UA was significant for relative HGS and LGS, but not for absolute HGS. Absolute HGS is confounded by anthropometric factors, whereas relative HGS (adjusted for body size) is recognized as a superior indicator of muscle strength and a more robust predictor of adverse outcomes like frailty, disability, and mortality (57). Our findings imply that UA, as a biologically active metabolite, may influence muscle health more directly by affecting intrinsic tissue quality, metabolic efficiency, and functional reserve, rather than solely diminishing maximal contractile force (40, 41). From another perspective, this underscores the importance of using multiple indicators (e.g., absolute values, relative values, and clinical diagnostic thresholds) in muscle aging and metabolic research to capture physiological information across different dimensions.

The present studyhas several notable strengths. First, we adopted an explicit sex-stratified analytical strategy to uncover the sex-specific mediating role of serum uric acid (UA) in the association between dietary diversity and muscle strength. UA significantly mediated the beneficial effect of dietary diversity on muscle strength in males, whereas the null findings in females may reflect a complex association pattern that could not be fully captured by linear mediation models. Within this stratified framework, restricted cubic spline analyses identified a male-specific UA threshold of approximately 363 μmol/L, which may serve as a potential reference for early risk stratification. Overall, this analytical approach underscores the necessity of accounting for sex-specific metabolic mechanisms in nutritional epidemiology, rather than assuming uniform effects across all participants. Second, the use of multidimensional muscle strength indicators provided a comprehensive phenotypic characterization, enhancing the clinical relevance of our findings. Furthermore, the comprehensive adjustment for a wide range of confounders, including the incorporation of estimated glomerular filtration rate (eGFR) as a continuous covariate, substantially improves the internal validity of our effect estimates. Beyond methodological advantages, this study was based on a community-based urban older adult cohort​ in central China under rapid urbanization, supplementing localized epidemiological data for Chinese older populations.

Several limitations should be acknowledged. First, the cross-sectional design cannot support causal conclusions. Though the pathway “dietary diversity → UA → muscle strength” holds biological plausibility, reverse and bidirectional relationships remain a major confounding concern. Specifically, reduced muscle strength may curtail daily physical activity and reshape dietary intake, disrupting metabolic profiles (32, 58) such as higher serum uric acid—a bidirectional association well-documented in older adults with sarcopenia or frailty (59, 60). Hence, we cannot reliably distinguish the temporal sequence of these correlated variables. Second, dietary assessment via FFQ is subject to recall bias, particularly in older adults. Moreover, the DDS—based solely on consumption frequency—captures dietary breadth but not portion sizes or nutrient density. Consequently, it cannot resolve purine variations from specific protein sources or culinary practices, a common trade-off in large-scale epidemiological studies. Third, single-time-point UA measurement, despite standardized fasting sampling, cannot capture long-term exposure due to short-term biological variability. This potential non-differential misclassification may attenuate true associations. Fourth, the study sample was restricted to urban-dwelling older adults in Hefei, Anhui Province, which may limit the generalizability of our findings to seniors living in other regions or rural areas. Moreover, male participants with UA < 300 μmol/L were scarce and unevenly distributed in our dataset, leading to wide confidence intervals and insufficient statistical power to draw solid conclusions about the antioxidant effects of low uric acid levels. Finally, although we adjusted for numerous covariates, residual confounding from unmeasured factors (e.g., genetic polymorphisms or specific bioactive food components) may persist. Despite these limitations, this study is the first to identify UA as a significant mediator linking dietary diversity to muscle strength among urban community-dwelling older men, advancing the mechanistic understanding of this relationship. The findings provide direct empirical support for dietary interventions aimed at improving metabolic health and preserving muscle function, which is particularly relevant for aging populations and individuals with high metabolic risk. Future research should employ longitudinal cohorts to track trajectories of UA levels and muscle strength, thereby generating more robust evidence to inform the development of targeted nutritional and metabolic intervention strategies.

## 5 Conclusion

In summary, this study demonstrates that, among urban community-dwelling older adults, dietary diversity is associated with muscle strength both directly and indirectly, with the latter mediated by serum uric acid (UA) levels. Notably, these associations—particularly the mediating role of UA—were significantly modified by sex, underscoring a complex, sex-specific metabolic pattern rather than a uniform mechanism. These findings carry dual implications. From a translational perspective, these findings suggest that maintaining optimal UA levels, particularly in older men, warrants attention as a potential target for future interventional studies aimed at preserving muscle strength. From a public health perspective, it reinforces the importance of promoting dietary diversity, advocating it not only as a means to ensure comprehensive nutrient intake but also as a strategic approach to improving metabolic health and, consequently, preserving muscle strength and function.

## Data Availability

The datasets generated and analyzed during the current study are not publicly available due to privacy and ethical restrictions but are available from the corresponding author, Liang Ruan, upon reasonable request.

## Supplementary material

Supplementary Figure S1 Sensitivity analysis of the association between UA and LGS defined by the EWGSOP2 criteria, stratified by sex. **(A)** Total population, **(B)** Male, **(C)** Female. Restricted cubic spline plots depict the associations of serum uric acid (UA) with low handgrip strength (LGS) in the total population, males, and females. Models for the LGS groups were adjusted for age, sex, educational attainment, marital status, living alone, income, smoking status, alcohol consumption, physical activity, eGFR, multimorbidity and BMI. In the sex-stratified analyses, sex was used as a stratification variable, not a covariate. The solid lines represent the odds ratios. The dashed horizontal line indicates the null effect (OR = 1). The shaded areas denote 95% confidence intervals. P values for the overall association and for nonlinearity are shown within each panel.
