# Supplementary material for: Sex-specific mediating role of serum uric acid in the association between dietary diversity and muscle strength in urban community-dwelling older adults
Source: Front Public Health. 2026 Aug 18;14:1877222. doi: 10.3389/fpubh.2026.1877222 (PMC13529517; doi:10.3389/fpubh.2026.1877222)
Supplement: Supplementary file 1 [file Table_1.DOCX]

**Table S1: Correlation coefficients among health-related indicators in the total population.**

|  |  | DDS | HGS | Relative HGS | UA | TC | TyG | MAP | MS | Age | BMI | eGFR |
| --- | --- | --- | --- | --- | --- | --- | --- | --- | --- | --- | --- | --- |
| DDS | Correlation coefficient | 1.00 | 0.08** | 0.10** | -0.05* | 0.07** | -0.04 | -0.04 | -0.04 | -0.05* | -0.04 | 0.11** |
|  | p-value |  | < 0.001 | < 0.001 | 0.036 | 0.006 | 0.101 | 0.124 | 0.187 | 0.034 | 0.074 | < 0.001 |
| HGS | Correlation coefficient |  | 1.00 | 0.91** | 0.23** | -0.10** | -0.05* | 0.02 | 0.04 | -0.18** | 0.07** | 0.09** |
|  | p-value |  |  | < 0.001 | < 0.001 | < 0.001 | 0.036 | 0.416 | 0.147 | < 0.001 | 0.005 | < 0.001 |
| Relative HGS | Correlation coefficient |  |  | 1.00 | 0.15** | -0.07** | -0.14** | -0.06* | -0.06* | -0.15** | -0.31** | 0.08** |
|  | p-value |  |  |  | < 0.001 | 0.008 | < 0.001 | 0.021 | 0.017 | < 0.001 | < 0.001 | 0.002 |
| UA | Correlation coefficient |  |  |  | 1.00 | -0.07** | 0.13** | 0.07** | 0.48** | 0.05* | 0.17** | -0.11** |
|  | p-value |  |  |  |  | 0.003 | < 0.001 | 0.005 | < 0.001 | 0.042 | < 0.001 | < 0.001 |
| TC | Correlation coefficient |  |  |  |  | 1.00 | 0.20** | 0.03 | 0.52** | -0.15** | -0.06** | 0.00 |
|  | p-value |  |  |  |  |  | < 0.001 | 0.304 | < 0.001 | < 0.001 | 0.009 | 0.991 |
| TyG | Correlation coefficient |  |  |  |  |  | 1.00 | 0.09** | 0.63** | -0.03 | 0.26** | -0.06* |
|  | p-value |  |  |  |  |  |  | < 0.001 | < 0.001 | 0.22 | < 0.001 | 0.012 |
| MAP | Correlation coefficient |  |  |  |  |  |  | 1.00 | 0.52** | 0.06* | 0.20** | -0.13** |
|  | p-value |  |  |  |  |  |  |  | < 0.001 | 0.011 | < 0.001 | < 0.001 |
| MS | Correlation coefficient |  |  |  |  |  |  |  | 1.00 | -0.03 | 0.25** | -0.14** |
|  | p-value |  |  |  |  |  |  |  |  | 0.279 | < 0.001 | < 0.001 |
| Age | Correlation coefficient |  |  |  |  |  |  |  |  | 1.00 | -0.03 | -0.44** |
|  | p-value |  |  |  |  |  |  |  |  |  | 0.23 | < 0.001 |
| BMI | Correlation coefficient |  |  |  |  |  |  |  |  |  | 1.00 | 0.01 |
|  | p-value |  |  |  |  |  |  |  |  |  |  | 0.884 |
| eGFR | Correlation coefficient |  |  |  |  |  |  |  |  |  |  | 1.00 |
|  | p-value |  |  |  |  |  |  |  |  |  |  |  |

Note: Correlation coefficients (r) and corresponding *P*-values are displayed in the lower-triangular matrix (^*^*P* < 0.05, ^**^*P* < 0.01). Variable abbreviations are defined in the main text.

**Table S2: Correlation coefficients among health-related indicators in males.**

|  |  | DDS | HGS | Relative HGS | UA | TC | TyG | MAP | MS | Age | BMI | eGFR |
| --- | --- | --- | --- | --- | --- | --- | --- | --- | --- | --- | --- | --- |
| DDS | Correlation coefficient | 1.00 | 0.14** | 0.11** | -0.13** | 0.02 | -0.00 | -0.04 | -0.07 | -0.03 | 0.01 | 0.14** |
|  | p-value |  | < 0.001 | 0.003 | < 0.001 | 0.626 | 0.945 | 0.337 | 0.084 | 0.386 | 0.734 | < 0.001 |
| HGS | Correlation coefficient |  | 1.00 | 0.86** | -0.01 | 0.09* | 0.07 | 0.08* | 0.10* | -0.36** | 0.16** | 0.09* |
|  | p-value |  |  | < 0.001 | 0.71 | 0.023 | 0.052 | 0.048 | 0.015 | < 0.001 | < 0.001 | 0.015 |
| Relative HGS | Correlation coefficient |  |  | 1.00 | -0.08* | 0.08* | -0.09* | -0.04 | -0.06 | -0.25** | -0.34** | 0.09* |
|  | p-value |  |  |  | 0.033 | 0.034 | 0.021 | 0.147 | 0.081 | < 0.001 | < 0.001 | 0.028 |
| UA | Correlation coefficient |  |  |  | 1.00 | 0.08* | 0.14** | 0.08* | 0.58** | -0.05 | 0.14** | -0.25** |
|  | p-value |  |  |  |  | 0.037 | < 0.001 | 0.039 | < 0.001 | 0.245 | < 0.001 | < 0.001 |
| TC | Correlation coefficient |  |  |  |  | 1.00 | 0.25** | 0.04 | 0.57** | -0.10* | -0.01 | 0.04 |
|  | p-value |  |  |  |  |  | < 0.001 | 0.38 | < 0.001 | 0.014 | 0.792 | 0.270 |
| TyG | Correlation coefficient |  |  |  |  |  | 1.00 | 0.10** | 0.65** | -0.08* | 0.30** | 0.00 |
|  | p-value |  |  |  |  |  |  | 0.007 | < 0.001 | 0.048 | < 0.001 | 0.975 |
| MAP | Correlation coefficient |  |  |  |  |  |  | 1.00 | 0.52** | -0.05 | 0.24** | -0.04 |
|  | p-value |  |  |  |  |  |  |  | < 0.001 | 0.27 | < 0.001 | 0.306 |
| MS | Correlation coefficient |  |  |  |  |  |  |  | 1.00 | -0.11** | 0.30** | -0.11** |
|  | p-value |  |  |  |  |  |  |  |  | 0.004 | < 0.001 | 0.004 |
| Age | Correlation coefficient |  |  |  |  |  |  |  |  | 1.00 | -0.18** | -0.38** |
|  | p-value |  |  |  |  |  |  |  |  |  | < 0.001 | < 0.001 |
| BMI | Correlation coefficient |  |  |  |  |  |  |  |  |  | 1.00 | 0.02 |
|  | p-value |  |  |  |  |  |  |  |  |  |  | 0.697 |
| eGFR | Correlation coefficient |  |  |  |  |  |  |  |  |  |  | 1.00 |
|  | p-value |  |  |  |  |  |  |  |  |  |  |  |

Note: Correlation coefficients (r) and corresponding *P*-values are displayed in the lower-triangular matrix (^*^*P* < 0.05, ^**^*P* < 0.01). Variable abbreviations are defined in the main text.

|  |  | DDS | HGS | Relative HGS | UA | TC | TyG | MAP | MS | Age | BMI | eGFR |
| --- | --- | --- | --- | --- | --- | --- | --- | --- | --- | --- | --- | --- |
| DDS | Correlation coefficient | 1.00 | 0.09** | 0.14** | -0.04 | 0.10** | -0.09** | -0.03 | -0.03 | -0.05 | -0.08* | 0.10** |
|  | p-value |  | 0.001 | < 0.001 | 0.278 | 0.003 | 0.008 | 0.267 | 0.424 | 0.101 | 0.019 | 0.002 |
| HGS | Correlation coefficient |  | 1.00 | 0.86** | -0.01 | 0.13** | -0.01 | 0.00 | 0.05 | -0.35** | 0.00 | 0.08* |
|  | p-value |  |  | < 0.001 | 0.639 | < 0.001 | 0.607 | 0.932 | 0.148 | < 0.001 | 0.867 | 0.018 |
| Relative HGS | Correlation coefficient |  |  | 1.00 | -0.10** | 0.16** | -0.12** | -0.09** | -0.06 | -0.31** | -0.48** | 0.07* |
|  | p-value |  |  |  | 0.002 | < 0.001 | < 0.001 | 0.006 | 0.053 | < 0.001 | < 0.001 | 0.032 |
| UA | Correlation coefficient |  |  |  | 1.00 | -0.00 | 0.21** | 0.05 | 0.52** | 0.04 | 0.17** | -0.13** |
|  | p-value |  |  |  |  | 0.924 | < 0.001 | 0.156 | < 0.001 | 0.258 | < 0.001 | < 0.001 |
| TC | Correlation coefficient |  |  |  |  | 1.00 | 0.14** | 0.05 | 0.54** | -0.13** | -0.10** | -0.01 |
|  | p-value |  |  |  |  |  | < 0.001 | 0.279 | < 0.001 | < 0.001 | 0.002 | 0.856 |
| TyG | Correlation coefficient |  |  |  |  |  | 1.00 | 0.08* | 0.64** | 0.03 | 0.22** | -0.10** |
|  | p-value |  |  |  |  |  |  | 0.023 | < 0.001 | 0.486 | < 0.001 | 0.003 |
| MAP | Correlation coefficient |  |  |  |  |  |  | 1.00 | 0.55** | 0.12** | 0.20** | -0.16** |
|  | p-value |  |  |  |  |  |  |  | < 0.001 | < 0.001 | < 0.001 | < 0.001 |
| MS | Correlation coefficient |  |  |  |  |  |  |  | 1.00 | 0.03 | 0.21** | -0.17** |
|  | p-value |  |  |  |  |  |  |  |  | 0.443 | < 0.001 | < 0.001 |
| Age | Correlation coefficient |  |  |  |  |  |  |  |  | 1.00 | 0.05 | -0.42** |
|  | p-value |  |  |  |  |  |  |  |  |  | 0.177 | < 0.001 |
| BMI | Correlation coefficient |  |  |  |  |  |  |  |  |  | 1.00 | -0.02 |
|  | p-value |  |  |  |  |  |  |  |  |  |  | 0.633 |
| eGFR | Correlation coefficient |  |  |  |  |  |  |  |  |  |  | 1.00 |
|  | p-value |  |  |  |  |  |  |  |  |  |  |  |

**Table S3: Correlation coefficients among health-related indicators in females.**

Note: Correlation coefficients (r) and corresponding *P*-values are displayed in the lower-triangular matrix (^*^*P* < 0.05, ^**^*P* < 0.01). Variable abbreviations are defined in the main text.

**Table S4 Mediation analysis of serum uric acid (UA) in the DDS – muscle strength association: direct, indirect, and total effects, stratified by sex.**

| Outcome | Subgroup | ACME | |  | ADE | |  | Total effect | | Mediation (%) |
| --- | --- | --- | --- | --- | --- | --- | --- | --- | --- | --- |
|  |  | Estimate (95% CI) | *P*-value |  | Estimate (95% CI) | *P*-value |  | Estimate (95% CI) | *P*-value |  |
| HGS | Total population | 0.0045 (-0.0066, 0.0190) | 0.420 |  | 0.3608 (0.1665, 0.5570) | < 0.001 |  | 0.3654 (0.1711, 0.5580) | < 0.001 | 1.01 |
|  | Male | 0.0183 (-0.0197, 0.0667) | 0.329 |  | 0.5450 (0.1680, 0.9189) | 0.002 |  | 0.5633 (0.1894, 0.9318) | 0.003 | 2.88 |
|  | Female | 0.0003 (-0.0082, 0.0093) | 0.933 |  | 0.2269 (0.0128, 0.4391) | 0.040 |  | 0.2272 (0.0126, 0.4403) | 0.039 | 0.04 |
| Relative HGS | Total population | 0.0010 (0.0001, 0.0021) | **0.036** |  | 0.0157 (0.0067, 0.0248) | < 0.001 |  | 0.0167 (0.0078, 0.0257) | < 0.001 | 5.64 |
|  | Male | 0.0020 (0.0001, 0.0049) | **0.032** |  | 0.0188 (0.0016, 0.0358) | 0.029 |  | 0.0208 (0.0036, 0.0376) | 0.017 | 9.22 |
|  | Female | 0.0002 (-0.0009, 0.0014) | 0.728 |  | 0.0138 (0.0035, 0.0239) | 0.010 |  | 0.0140 (0.0037, 0.0242) | 0.010 | 1.23 |
| LGS | Total population | -0.0012 (-0.0029, -0.0000) | **0.049** |  | -0.0275 (-0.0449, -0.0101) | 0.002 |  | -0.0287 (-0.0461, -0.0113) | 0.001 | 3.79 |
|  | Male | -0.0031 (-0.0073, -0.0003) | **0.022** |  | -0.0309 (-0.0537, -0.0050) | 0.017 |  | -0.0341 (-0.0566, -0.0083) | 0.008 | 8.67 |
|  | Female | -0.0001 (-0.0013, 0.0013) | 0.998 |  | -0.0224 (-0.0456, -0.0001) | 0.048 |  | -0.0224 (-0.0458, -0.0000) | 0.050 | 0.01 |

Note: Data are presented as estimate (95% confidence interval) and *P*-value. ACME, average causal mediation effect (indirect effect via UA); ADE, average direct effect (direct effect of DDS on muscle strength independent of UA). The proportion mediated is calculated as (ACME / Total effect) × 100% and indicates the percentage of the total effect explained by the indirect pathway through UA. Models for the HGS and relative HGS groups were adjusted for age, sex, educational attainment, marital status, living alone, income, smoking status, alcohol consumption, eGFR, and physical activity. Models for the LGS group were further adjusted for multimorbidity and BMI. In the sex-stratified analyses, sex was used as a stratification variable, not a covariate. Variable abbreviations are defined in the main text.

| Variable | Model 1 | |  | Model 2 | |  | Model 3 | |
| --- | --- | --- | --- | --- | --- | --- | --- | --- |
|  | *OR* (95% CI) | *P*-value |  | *OR* (95% CI) | *P*-value |  | *OR* (95% CI) | *P*-value |
| DDS | 0.864 (0.800, 0.933) | **<0.001** |  | 0.866 (0.799, 0.938) | **<0.001** |  | 0.873 (0.801, 0.952) | **0.002** |
| UA | 1.002 (1.000, 1.003) | **0.003** |  | 1.001 (1.000, 1.003) | 0.064 |  | 1.002 (1.000, 1.003) | **0.014** |

**Table S5 Sensitivity analysis of the associations between dietary diversity, serum uric acid, and low grip strength defined by the EWGSOP2 criteria.**

**Note: Logistic regression for binary outcome (LGS), reported as OR (95% CI). Model 1: Crude unadjusted model. Model 2: Adjusted for age and sex. Model 3: Multivariable model adjusted for age, sex, education, marital status, living status, income, smoking, alcohol intake, physical activity, eGFR, multimorbidity and BMI. All variable abbreviations are defined in the main text.**

**Table S6 Mediation analysis of serum uric acid (UA) in the association between DDS and LGS defined by the EWGSOP2 criteria: direct, indirect, and total effects, stratified by sex.**

| Outcome | Subgroup | ACME | |  | ADE | |  | Total effect | | Mediation (%) |
| --- | --- | --- | --- | --- | --- | --- | --- | --- | --- | --- |
|  |  | Estimate (95% CI) | *P*-value |  | Estimate (95% CI) | *P*-value |  | Estimate (95% CI) | *P*-value |  |
| LGS | Total population | -0.0015 (-0.0035, -0.0002) | **0.013** |  | -0.0271 (-0.0476, -0.0079) | 0.003 |  | -0.0285 (-0.0492, -0.0092) | 0.001 | 5.03 |
|  | Male | -0.0028 (-0.0069, -0.0001) | **0.040** |  | -0.0279 (-0.0539, -0.0012) | 0.038 |  | -0.0307 (-0.0567, -0.0039) | 0.022 | 8.68 |
|  | Female | -0.0001 (-0.0012, 0.0012) | 0.999 |  | -0.0233 (-0.0520, -0.0002) | 0.047 |  | -0.0233 (-0.0521, -0.0001) | 0.049 | 0.01 |

Note: Data are presented as estimate (95% confidence interval) and *P*-value. ACME, average causal mediation effect (indirect effect via UA); ADE, average direct effect (direct effect of DDS on muscle strength independent of UA). The proportion mediated is calculated as (ACME / Total effect) × 100% and indicates the percentage of the total effect explained by the indirect pathway through UA. Models for the LGS groups were adjusted for age, sex, educational attainment, marital status, living alone, income, smoking status, alcohol consumption, physical activity, eGFR, multimorbidity and BMI. In the sex-stratified analyses, sex was used as a stratification variable, not a covariate. Variable abbreviations are defined in the main text.
